# Ischemic Nephropathy Following Occlusion of Abdominal Aortic Aneurysm Graft: A Case Report

**DOI:** 10.7759/cureus.13799

**Published:** 2021-03-10

**Authors:** Kevin Dao, Pooja Patel, Erin Pollock, Andrew Mangano, Kiranpreet Gosal

**Affiliations:** 1 Internal Medicine, Grand Strand Medical Center, Myrtle Beach, USA

**Keywords:** abdominal aortic aneurysms, ischemic nephropathy, end-stage renal disease (esrd), renal artery stenosis, contrast induced nephropathy, resistant hypertension, flash pulmonary edema

## Abstract

In this report, we present a case of a 55-year-old female with a past medical history of abdominal aortic aneurysm (AAA) graft, femoral-femoral bypass graft, questionable history of chronic kidney disease (CKD), abdominal hernia repair, alcoholic pancreatitis, chronic abdominal pain on opioids, and tobacco abuse who presented with acute on chronic abdominal pain with an unexplained rise of creatinine and anuria. The patient was found to have complete occlusion of AAA graft and was determined to have ischemic nephropathy (IN).

## Introduction

Ischemic nephropathy (IN) is defined as the progressive reduction of glomerular filtration rate (GFR) as a result of diminished renal blood flow [1]. Etiologies include renovascular occlusive diseases (RVD) such as atherosclerotic renal artery stenosis (RAS) and, less commonly, fibromuscular dysplasia [2]. Some findings that indicate renovascular disease are severe hypertension (HTN) that is resistant to treatment, acute rise in serum creatinine following the administration of angiotensin-converting enzyme (ACE) inhibitors or angiotensin receptor blockers (ARBs), and flash pulmonary edema [3]. Doppler ultrasonography (US) is typically the first tool used to evaluate for renal vascular disease, but this examination may be time-consuming and the results of the ultrasound examination are operator-dependent [1,4]. We present a case of IN secondary to occlusion of abdominal aortic aneurysm (AAA) graft to highlight the difficulty in its diagnosis and identify opportunities for the selection of appropriate imaging techniques.

This article was previously presented as a virtual poster at the 2020 PeeDee Local Chapter of the Society of Hospital Medicine on November 14, 2020, and was awarded second place.

## Case presentation

A 55-year-old female with a past medical history of AAA graft, femoral-femoral bypass graft on clopidogrel, questionable history of chronic kidney disease (CKD), recent diagnosis of posterior reversible encephalopathy syndrome (PRES), uncontrolled HTN, abdominal hernia repair, alcoholic pancreatitis, cholecystectomy, chronic abdominal pain on opioids, and tobacco abuse presented with a three-day history of acute on chronic abdominal pain with associated nausea, vomiting, constipation, and decreased urine output. She reported chronic mild diffuse abdominal tenderness with right-sided abdominal tenderness developing suddenly, described as sharp pain radiating to her back. The patient had gone to multiple hospitals for treatment; however, she had been turned away for concern about drug-seeking behavior. The patient was taking high doses of hydromorphone (4 mg three times a day) for her abdominal pain and diazepam 5 mg daily as needed for anxiety for the last several months. She denied trauma, fever, chills, diarrhea, dysuria, and hematuria.

**Figure 1:**
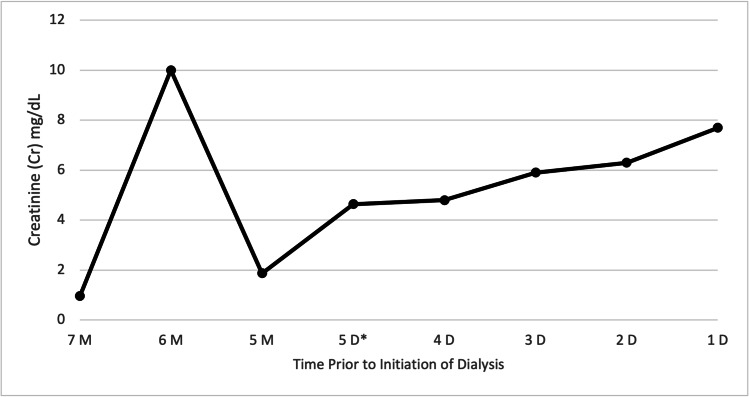
Creatinine trend prior to the initiation of dialysis M: months prior to initiation of dialysis; D: days prior to initiation of dialysis. D*: day of admission

On admission, the patient was afebrile, with a heart rate of 92 beats per minute. Blood pressure was elevated at 173/93 mmHg and oxygen saturation was normal on room air. Physical examination was significant for abdominal surgical scars and right flank tenderness. No rebound tenderness was present. Laboratory workup was significant for a white blood cell (WBC) count of 13.7 K/mm^3^ with a neutrophil predominance (80.8%), hemoglobin (Hgb) of 17.9 gm/dl, platelets (PLT) of 368 K/mm^3^, anion gap of 17 mEq/L, creatinine of 4.8 mg/dL, and GFR of 9 mL/min/1.73 m^2^. Lactic acid, albumin, lipase, and lipid panels were unremarkable. Seven months prior to her presentation, the patient's kidney functions had been within normal limits, but a month after, she had been noted to have fluctuating creatinine and stage 4 CKD, which can be seen in Figure 1. Her acute kidney injury (AKI) had been attributed to HTN and PRES, which had improved with conservative management. She had been discharged at that time with a recommendation for outpatient and nephrology follow-up.

**Figure 2:**
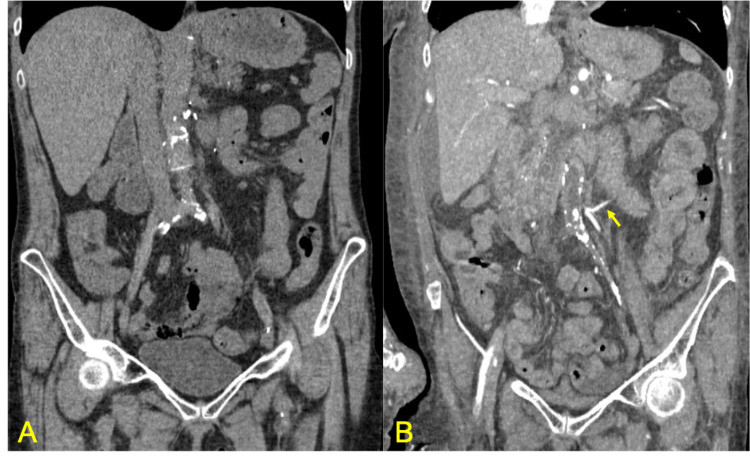
CT vs. CTA of abdomen and pelvis A: non-contrast CT of the abdomen and pelvis showed stable surgical changes and limited view of the vasculature. B: CT angiography (CTA) of the patient's abdomen and pelvis showed complete occlusion of abdominal aorta aneurysm graft and poor vasculature. A collateral vessel called the arc of Riolan (arrow) is shown, which is formed between the proximal superior mesenteric artery and the inferior mesenteric artery in the setting of severe vascular occlusion [5] CT: computed tomography

On presentation in the emergency room, imaging with contrast was not performed due to her acute renal failure and concerns for contrast-induced nephropathy (CIN). Alternatively, the patient underwent a non-contrast CT of her abdomen and pelvis, which showed stable calcifications in the central abdomen favoring chronic pancreatitis, severe atrophy of left kidney greater than right, right renal vascular calcifications, and stable postsurgical changes from cholecystectomy and femoral-femoral bypass surgery (Figure 2A). She was started empirically on ceftriaxone for suspected pyelonephritis versus other abdominal sources of infection such as abscess, intravenous fluids, ondansetron, morphine, and fentanyl. On day two, the patient’s symptoms persisted with increased leukocytosis of 17.1 K/mm^3^ with worsening creatinine and GFR. She reported anuria overnight and was transitioned to piperacillin/tazobactam for empiric treatment of an abdominal source of infection. Nephrology and general surgery were consulted for further evaluation. Due to unremarkable imaging and labs, general surgery suspected that the patient’s symptoms were likely chronic due to a known history of chronic pancreatitis.

Nephrology performed further workup of fluctuating creatinine. Urinalysis was obtained by straight catheterization, which was negative for infection and red blood cells but had a prominent urine protein of 100 mg/dL. Urine protein creatinine ratio was calculated to be 13.6 g/day (normal level: <0.2 g/day, nephrotic range: >3.5 g/day) and fractional excretion of sodium (FENa) was calculated to be 0.4%. Complement C3, complement C4, cytoplasmic anti-neutrophil cytoplasmic antibodies (C-ANCA), perinuclear anti-neutrophil cytoplasmic antibodies (P-ANCA), serum protein electrophoresis (SPEP), and urine protein electrophoresis (UPEP) were within normal limits. On day three, the patient reported persistent abdominal pain requiring hydromorphone 4 mg four times a day. Intravenous fluids were continued due to potential pre-renal etiology. Leukocytosis improved from 17.1 K/mm^3^ to 12.3 K/mm^3^. Renal Doppler was performed, which showed a small left kidney and increased echogenicity of the right kidney consistent with medical renal disease with no hydronephrosis or abscesses. Imaging was discussed with radiology who reported adequate flow seen on renal Doppler; however, they were unable to accurately read it due to poor technical study. On day four, the patient began to have mild anasarca and shortness of breath with persistent anuria. On day five, intravenous fluids were discontinued due to increased work of breathing. CT of her chest was obtained, which showed bilateral moderate pulmonary effusions, progressive bilateral lower lobe consolidations, and new development of bilateral ground-glass infiltrations consistent with potential flash pulmonary edema. A tunneled catheter was placed and dialysis was initiated for volume overload. Due to unexplained creatinine rise and persistent abdominal pain, CT angiography (CTA) of abdomen and pelvis was obtained, which showed occluded femoral-femoral bypass graft, occlusion of the aorta to the right and left common femoral artery bypass graft (patent one year ago), and impaired renal perfusion due to complete occlusion of the aorta just after the superior mesenteric artery (SMA) branches off. There were minimal collateral vessels to the kidneys. Celiac artery and SMA were patent with the reconstitution of left common femoral artery with collateral vessels (Figure 2B). The inferior mesenteric artery was reconstituted via a collateral vessel called the arc of Riolan (Figure 2B; arrow). Vascular surgery was consulted for potential surgical intervention. The patient was placed on a heparin drip and transferred to the intensive care unit (ICU). Due to the significance of coagulopathy and limited renal function requiring dialysis, it was determined that the risk of surgical intervention outweighed the benefits. Hence, she was treated with supportive care and appropriate pain management. Her heparin drip was discontinued and apixaban was added to her clopidogrel. She was transferred out of the ICU in stable condition and with relief knowing the origin of her pain. She was discharged for outpatient follow-up and routine hemodialysis.

## Discussion

Progressive kidney dysfunction and HTN are the predominant features of IN [1]. Patients who have a progressive reduction in GFR with unknown etiologies should be evaluated for renovascular disease, which manifests as an acute rise in serum creatinine after angiotensin blockade, resistant malignant HTN, new onset of HTN (more likely atherosclerotic disease if the patient is more than 50 years in age; more likely due to fibromuscular dysplasia if the patient is less than 50 years), fluctuation of creatinine due to volume status, flash pulmonary edema, congestive heart failure, and deterioration of renal function after placement of endovascular aortic stent graft [1,6,7]. Of note, 60-90% of IN cases are due to atherosclerosis, 10-30% are due to fibromuscular dysplasia, and less than 10% are due to vasculitis, thromboembolic disease, and other causes [6]. The exact prevalence of IN is unknown, but it has been estimated to be responsible for end-stage renal disease (ESRD) in approximately 5-22% of patients who are more than 50 years old [1]. Other risk factors for atherosclerosis include age of more than 50 years, hyperlipidemia, and tobacco use [6]. Early detection and medical therapy are important as a delay in diagnosis could lead to worsening of the disease, yet studies have shown that kidney function may deteriorate even after renal vascularization with no apparent mortality benefit [8].

To evaluate for IN, labs should include serum creatinine and urine protein-creatinine ratio (mild to moderate degree of proteinuria that is usually not in nephrotic range) to assess renal dysfunction, a urinalysis to rule out glomerulonephritis, and serologic studies to rule out rheumatologic disease, including antinuclear antibodies, C3, C4, and antinuclear cytoplasmic antibodies [6]. Patients with concerns of IN should be considered for renal arteriography, which is considered the gold standard; however, duplex US has been the initial test of choice due to low costs, accessibility, and reported high sensitivities and specificities (approximately 90%). Although many use renal Dopplers as a screening tool for RAS and IN, ultrasounds are operator-dependent, with failure rates as high as 20% [9]. CTA and gadolinium-enhanced magnetic resonance angiography (MRA) have the highest diagnostic probability, as per a meta-analysis of 55 studies comparing CTA, MRA, US, and captopril scintigraphy [1,10]. If clinical suspicion is still high, CTA and MRA should be ordered regardless of kidney function. In a retrospective study of more than 12,000 patients, the AKI reportedly seemed to be independent of contrast exposure even in patients with CKD stage 4 and higher [11]. Other studies have shown that the incidence of acute adverse events from contrast is similar to patients who are not exposed, and the incidence of CIN may be overestimated [12,13]. MRA is another option; however, this test is typically avoided in patients with renal insufficiency due to adverse effects such as gadolinium nephrogenic systemic fibrosis (NSF) [6]. Some studies have shown that adverse effects of gadolinium-based contrast agents may be overestimated as well and have been shown to cause NSF in only 0.07% of CKD stage 4 and stage 5 patients [14]. If there are high concerns for severe renal damage from imaging, peri-procedural hydration, low-osmolar or iso-osmolar contrast, and reducing the load of contrast seem to be most beneficial for patients at risk of CIN. If the fluid overload is an issue, there are mixed studies that show pharmacologic agents such as acetylcysteine and fenoldopam may be of some benefit in reducing renal injury [15,16].

Treatment of RAS and IN include management of HTN, cholesterol, heart failure, pulmonary edema, and prevention of nephropathy. Angiotensin blockers are recommended in patients with early ischemic renal disease, which may improve cardiovascular mortality by up to 10%; however, these are of limited utility due to acute creatinine rise and hyperkalemia [17]. Patients can have a creatinine rise of more than 30% of baseline creatinine and this medication should be discontinued in the setting of continued renal dysfunction [6]. Statin and antiplatelet therapy have also shown improvement in mortality and should be started in patients with RAS or concerns of atherosclerosis [18]. Patients who have failed medical treatment for resistant HTN and those who have recurrent flash pulmonary edema or heart failure should be considered for percutaneous transluminal angioplasty (PTRA) with or without stent placement. If kidney deterioration is chronic, kidney size is less than 8.0 cm, or if the resistive index (US-calculated measurement of renal blood flow) is equal to or greater than 0.80, patients tend to have little improvement in clinical status and renal function after revascularization [2]. Surgical revascularization is another option; however, in a small randomized trial comparing angioplasty with surgery, improvements in HTN and renal functions were found to be similar, supporting nonsurgical intervention as the first-line treatment. In addition, patients who undergo surgical revascularization may have in-hospital mortality as high as 10% [18]. Some reports have shown that more than 35% of patients with renovascular diseases will require dialysis and have an accelerated mortality. In patients who require renal replacement therapy due to renovascular disease, a 25-month median survival and a five-year survival rate of 18% have been reported [2].

## Conclusions

When dealing with patients with known severe vascular disease, clinicians should maintain a high index of suspicion for IN, especially in the setting of unexplained creatinine rise, HTN, and signs of volume overload. Patients requiring chronic narcotics and those who are suspected of narcotic abuse should be carefully examined for organic causes of symptoms. Quality of renal Dopplers are operator-dependent and further investigation with CTA or MRA is warranted if clinical suspicion remains high for RAS or IN even in the setting of CKD. Further studies are required to evaluate appropriate imaging modalities in patients with severe coagulopathy and complicated anatomy.
